# PTEN negatively regulates mTORC2 formation and signaling in grade IV glioma via Rictor hyperphosphorylation at Thr1135 and direct the mode of action of an mTORC1/2 inhibitor

**DOI:** 10.1038/oncsis.2016.34

**Published:** 2016-05-30

**Authors:** K Bhattacharya, S Maiti, C Mandal

**Affiliations:** 1 Cancer Biology and Inflammatory Disorder Division, Council of Scientific and Industrial Research (CSIR)-Indian Institute of Chemical Biology, Kolkata, India

## Abstract

To investigate the role of PTEN (phosphatase and tensin homolog) in mammalian target of rapamycin complex 2 (mTORC2) signaling in glioblastoma multiforme (GBM), we found higher activation of mTORC2 in PTEN^mu^ cells, as evidenced by enhanced phosphorylation of mTOR (Ser2481), AKT (Ser473) and glycogen synthase kinase 3 beta (GSK3β) (Ser9) as compared with PTEN^wt^ cells. In addition, PTEN^wt^ cells upon PTEN depletion showed mTORC2 activation. The reduced mTORC2 signaling in PTEN^wt^ cells was related to higher Rictor phosphorylation at Thr1135 residue. Phosphorylation of Rictor at Thr1135 inhibited its association with mTORC and thus there was a reduction in mTORC2 complex formation. In addition, PTEN^wt^ cells expressing mutated Rictor in which Thr1135 was substituted with alanine, showed enhanced mTORC2 formation and signaling. This enhanced mTORC2 signaling promoted inactivation of GSK3β. Thus, we established the reciprocal activation of mTORC2 and GSK3β in GBM. To the best of our knowledge, this is the first report describing role of PTEN in mTORC2 formation by promoting Rictor phosphorylation (Thr1135) in GBM. Furthermore, the drug sensitivity of mTORC2 was evaluated. A newly identified carbazole alkaloid, mahanine, showed cytotoxicity in both PTEN^mu^ and PTEN^wt^ cells. It inhibited both mTORC1/2 and AKT completely in PTEN^mu^ cells, whereas it inhibited only mTORC1 in PTEN^wt^ cells. Cytotoxity and AKT-inhibitory activity of the mTORC1/2 inhibitor was increased either by depleting PTEN or in combination with phosphatidylinositol 3 kinase inhibitors in PTEN^wt^ cells. In contrast, depletion of Rictor decreased the cytotoxicity of the mTORC1/2 inhibitor in PTEN^mu^ cells. Thus, PTEN has an important role in mTORC2 formation and also influences the effectiveness of an mTORC1/2 inhibitor in GBM.

## Introduction

Mammalian target of rapamycin (mTOR), a serine/threonine (Ser/Thr) kinase protein, has a central role in cell growth and proliferation.^1, 2^ mTOR complex 1 (mTORC1) and complex 2 (mTORC2) are two functionally distinct complexes having some common subunits.^3, 4, 5^ In addition, mTORC1 contains two more specific subunits namely regulatory-associated protein of mammalian target of rapamycin (Raptor) and PRAS40.^6, 7, 8, 9, 10^ Rapamycin-insensitive companion of mTOR (Rictor), mSin1 and protor1/2, are exclusive partners of mTORC2.^11, 12, 13^ The binding of Rictor or Raptor to mTOR is mutually exclusive in a certain cellular scenario.

mTORC1 promotes protein translation through activation of S6K1, inhibition of 4E-BP1 and enhancement of RNA translation via S6 ribosomal protein.^2, 14^ mTORC2 can specifically phosphorylate AKT at the Ser473 and take part in cell proliferation, regulation and cytoskeletal reorganization.^15, 16^ Rapamycin and its analogs (rapalogs) are used for treatment of cancers as mTORC1 inhibitors. However, inhibition of mTORC1 induces the activation of other survival pathways and thus reduces the efficacy of rapalogs.^17, 18, 19^ Therefore, targeting mTORC2 may possibly have better therapeutic values.^20^ Accordingly, exploration of new improved mTORC1/2 inhibitors is of great interest.

PTEN (phosphatase and tensin homolog), a tumor suppressor protein, is often inactivated in cancers.^21^ Cellular cross-talk between PTEN and mTORC1 via the phosphatidylinositol 3 kinase (PI3K)/AKT/mTOR pathway is often deregulated and enhances the malignancy.^22, 23^ Although the role of mTORC1 is well characterized, the function and regulation of mTORC2 is still poorly understood.

**Figure 1:**
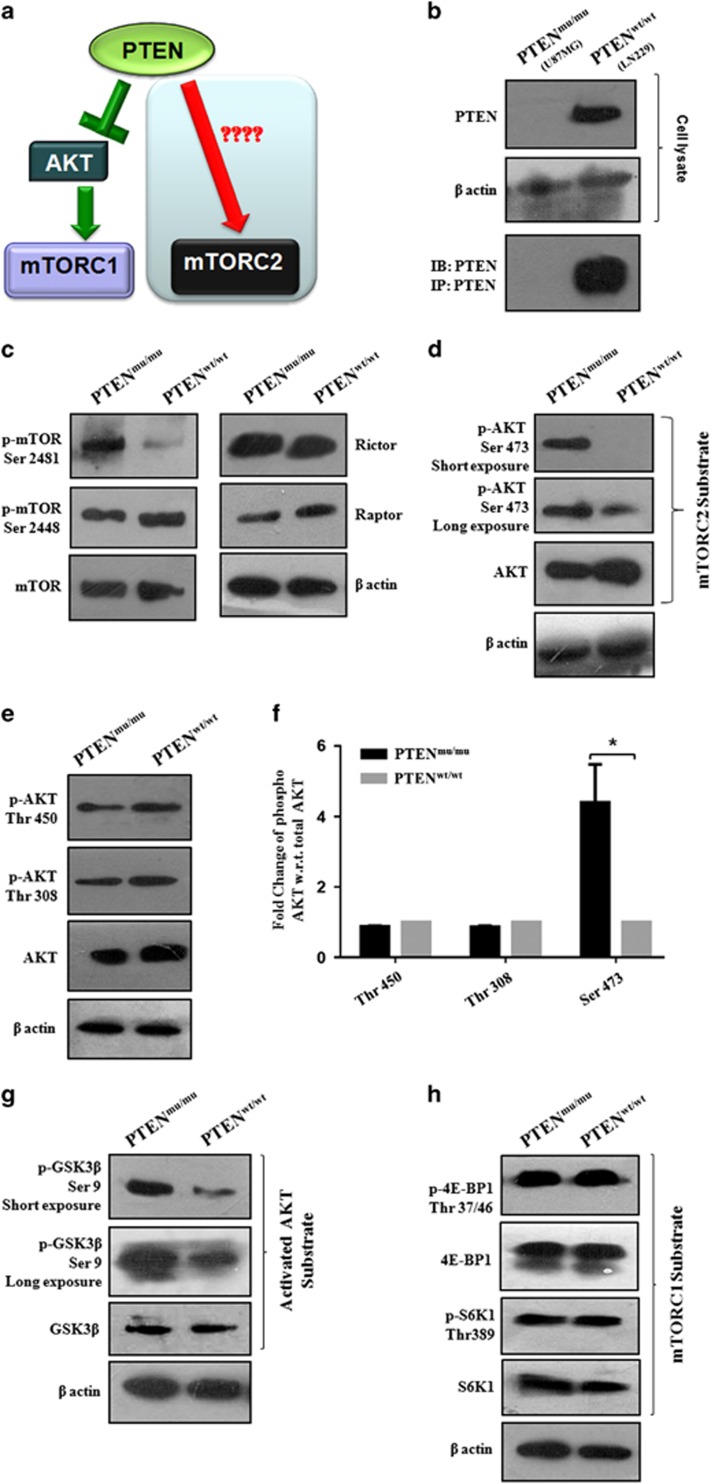
Differential activation of mTORC1 and mTORC2 in PTEN^wt^ and PTEN^mu^ GBM cells along with downstream signaling. (**a**) Schematic representation of proposed hypothesis: role of PTEN in mTORC2 signaling pathways. (**b**–**h**) PTEN^mu^ and PTEN^wt^ cells were harvested and lysed and kept in phosphate-buffered saline (PBS) with protease and phosphatase inhibitors. Protein concentrations were measured by the Bradford's protein assay reagent. Proteins (50-100 μg) from each sample were resolved in sodium dodecyl sulfate–polyacrylamide gel electrophoresis (SDS–PAGE; 5–12%) followed by electrotransfer into nitrocellulose/polyvinylidene difluoride (PVDF) membrane and probed with primary antibodies (1:1000–1:2000) and incubated overnight at 4 ^o^C. Then the membrane was washed with Tween-20 (0.1%) containing Tris-buffered saline (TBS) and incubated with horseradish peroxidase-conjugated secondary antibodies (1:1000). They were washed and target proteins were identified by the West Pico chemiluminiscence detector system or Biorad ChemiDoc MP System (Bio-Rad). Developed bands of corresponding proteins were densitometrially analyzed by ImageJ software. (**b**) Level of PTEN in PTEN^mu^ and PTEN^wt^ cells. (**c**) Status of mTOR, p-mTOR Ser2448, p-mTOR Ser2481, Rictor and Raptor. (**d**) AKT, the downstream substrate of mTORC2 was highly phosphorylated at Ser473 in PTEN^mu^ cells. (**e**) Status of AKT phosphorylation at the Thr450 and Thr308 positions. (**f**) Densitometric analysis showed phosphorylation of AKT at the Thr450, Thr308 and Thr 473 sites. Values were normalized against the relative expression of β-actin and total AKT, quantified by ImageJ software. Each value was the mean±s.d. of three independent experiments. *P<0.05, significant difference between two test groups. (**g**) Status of Ser9 phosphorylation of GSK3β, the substrate of activated AKT. (**h**) mTORC1 activity readout as identified by phosphorylation of S6K1 and 4E-BP1. β-Actin served as loading control for all the western blots.

Glioblastoma multiforme (GBM) is a grade IV brain tumor with higher mortality rate. *PTEN*, *EGFR*, *TP53*, *CDKN2A*, *CDK4* and *LOH* are several frequently mutated genes.^24^ PTEN mutations are frequently involved with this aggressive cancer, our initial aim was to decipher the regulation of mTORC2 with respect to PTEN wild-type (PTEN^wt^) vs mutated conditions (PTEN^mu^; Figure 1a). This information may help us to search for an effective therapeutic strategy for disease management. Earlier, we identified a non-toxic novel carbazole alkaloid (mahanine), which induced apoptosis in several cancers including GBM.^25, 26, 27, 28, 29, 30, 31, 32^ Therefore, our next aim was to identify the mode of activity of mahanine as a possible mTOC1/2 inhibitor and enhance the sensitivity based on cellular existence of mTORC1/2.

Here we provide evidence for PTEN-mediated regulation of mTORC2 in GBM. We showed that PTEN mutations lead to reduced phosphorylation of Rictor at Thr1135, which in turn promotes enhanced mTORC2 formation and downstream signaling. However, higher phosphorylation of Rictor at Thr1135 leads to the reduction of mTORC2 formation in PTEN^wt^ cells. Furthermore, we confirmed a positive correlation between enhanced mTORC2 formation and sensitivity toward mahanine in PTEN-mutated cells. Mahanine also inhibited mTORC1 activity and may thus be considered as a potential mTORC1/2 inhibitor.

## Results

### Differential mTORC1 and mTORC2 activity in PTEN^wt^ and PTEN^mu^ cells

To understand the impact of functional PTEN in mTORC2 formation and downstream signaling, we selected PTEN^mu^ (U87MG) and PTEN^wt^ (LN229) GBM cells and the status of PTEN was verified (Figure 1b). Next, we examined the status of specific phosphorylation of mTOR complexes. Active mTORC2-specific phosphorylation of mTOR at Ser2481 was higher in PTEN^mu^ cells (Figure 1c). In contrast, mTORC1-specific phosphorylation at Ser2448 was almost comparable in PTEN^wt^ and PTEN^mu^ cells. However, Rictor and Raptor, two major mTORC2 and mTORC1-associated proteins were very similar in both the cells. This result indicates probable activation of mTORC2 because of PTEN mutation.

To confirm this upstream activation of mTORC2 in PTEN^mu^ cells, we examined the phosphorylation status of a few downstream molecules. Ser473 of AKT is a selective substrate of mTORC2. We observed enhanced expression of phospho-AKT at Ser473 in PTEN^mu^ cells (Figure 1d). As mTORC2 is not known to influence the Thr308 of AKT, we detected almost comparable levels of phosphorylation in both PTEN^wt^/PTEN^mu^ cells (Figure 1e). In addition, no significant change of AKT phosphorylation at the Thr450 was found. Densitometric analysis of three phosphorylation sites of AKT revealed that only Ser473 was ~5-fold enhanced in PTEN^mu^ cells relative to PTEN^wt^ (Figure 1f). This observation strongly suggested that PTEN mutation may have a significant role in regulating mTORC2 activity, which in turn enhanced the phosphorylation of its substrate AKT at the Ser473.

To confirm the activation of AKT through its Ser473 phosphorylation, next we focused on GSK3β. Activated AKT phosphorylates GSK3β at the Ser9 position and induces its inactivation. Phosphorylation of GSK3β at Ser9 was higher in PTEN^mu^ cells, indicating the sequential signaling of activated mTORC2 and AKT and subsequent inactivation of GSK3β (Figure 1g). This result suggested the reciprocal relationship of mTORC2 and GSK3β. In contrast, mTORC1 activity was similar in both PTEN^mu^/PTEN^wt^ cells as judged by the equivalent phosphorylation of S6K1 at Thr389 and 4E-BP1 at Thr37/46 (Figure 1h).

### Knockdown of PTEN enhances mTORC2 signaling in PTEN^wt^ cells

**Figure 2:**
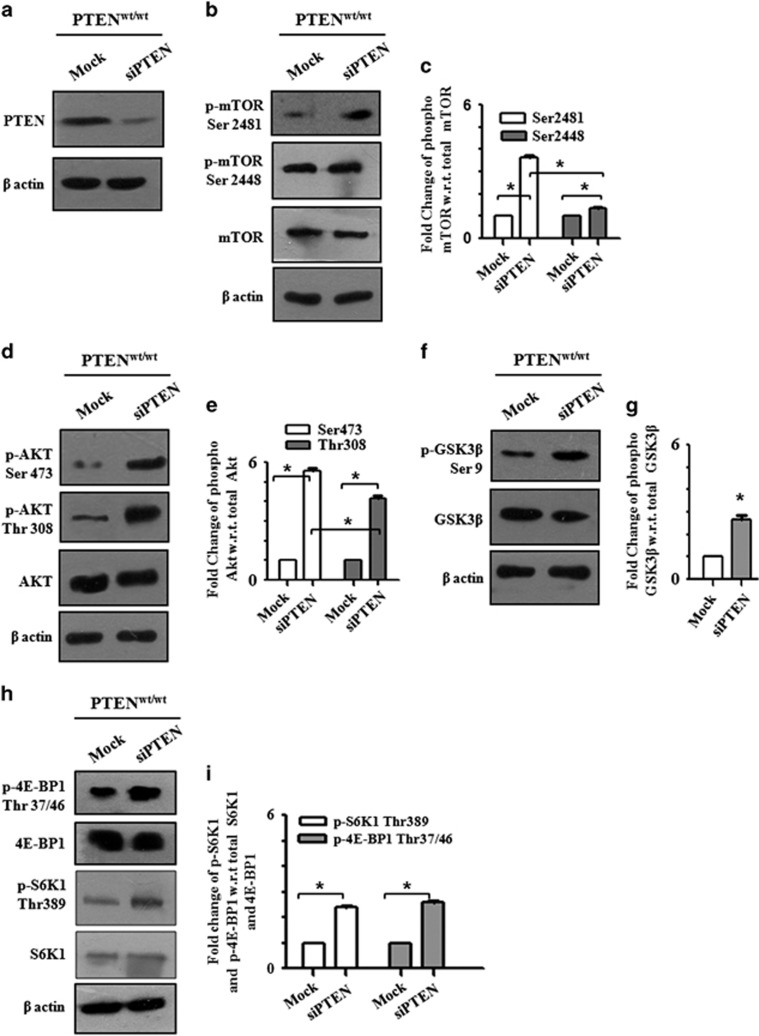
Knockdown of PTEN from PTEN^wt^ GBM cells enhances the mTORC2 signaling pathway. PTEN^wt^ cells were transfected with targeted siRNA against PTEN. After 24 h of transfection, cell lysates were subjected to western blot analysis. (**a**) Status of PTEN after siRNA transfection in PTEN^wt^ cells. (**b**) Expression of mTOR and p-mTOR were determined by specific antibodies. (**c)** Densitometric analyses mTOR and p-mTOR blots. (**d**) Phosphorylation of AKT at Ser473 and Thr308 residues with respect to total AKT. (**e**) Densitometry values of AKT at Ser473. (**f**) Status of GSK3β phosphorylation at Ser9 residue. (**g**) Relative densitometry value of GSK3β Ser9 western blot. (**h**) mTORC1 activity readout judged by the phosphorylation of its downstream substrate 4E-BP1 and S6K1. (**i**) Fold change of p-S6K1 and p-4E-BP1 showed with relative densitometry analysis. β-Actin was served as loading control in all set of experiments. All the densitometric analysis was done by ImageJ software and normalized against the expression of β-actin and respective total protein level. The protein levels from three independent experiments were quantified and presented as mean±s.d. Asterisk indicates significant difference when compared with the control (*P*<0.05).

To further confirm the role of PTEN in mTORC2 activity, we transiently knocked down PTEN using targeted short interfering RNA (siRNA) in PTEN^wt^ cells (Figure 2a), which exhibited ~4-fold increase in mTORC2-specific phosphorylation of mTOR at Ser2481 (Figures 2b and c), demonstrated by the western blot analysis. This result supported our initial observation in PTEN^mu^ cells (Figure 1), confirming the activation of mTORC2 in the absence of wild-type PTEN. The downstream substrate of mTORC2 (Ser473 of AKT) was also highly phosphorylated upon PTEN depletion (Figures 2d and e) and the inhibitory Ser9 phosphorylation of GSK3β was also enhanced (Figures 2f and g). These findings reconfirmed the reciprocal activation profile of mTORC2 and GSK3β. We have also observed the enhanced phosphorylation at Thr308 (substrate of PDK1) of AKT upon PTEN knockdown (Figures 2d and e). This served as a positive control for PTEN deactivation and subsequent activation of the PI3K pathway, which in turn activated mTORC1 as evidenced by increased mTOR phosphorylation at Ser2448 (Figures 2b and c). As mTORC1 activity is regulated by the PI3K pathway, therefore we next studied the phosphorylation of S6K1 and 4E-BP1. We observed hyperphosphorylation of S6K1 at Thr389 and 4E-BP1 at Thr37/46 after knockdown of wild-type PTEN (Figures 2h and i).

### Identification of Rictor as the target molecule of PTEN for mTORC2 signaling

**Figure 3:**
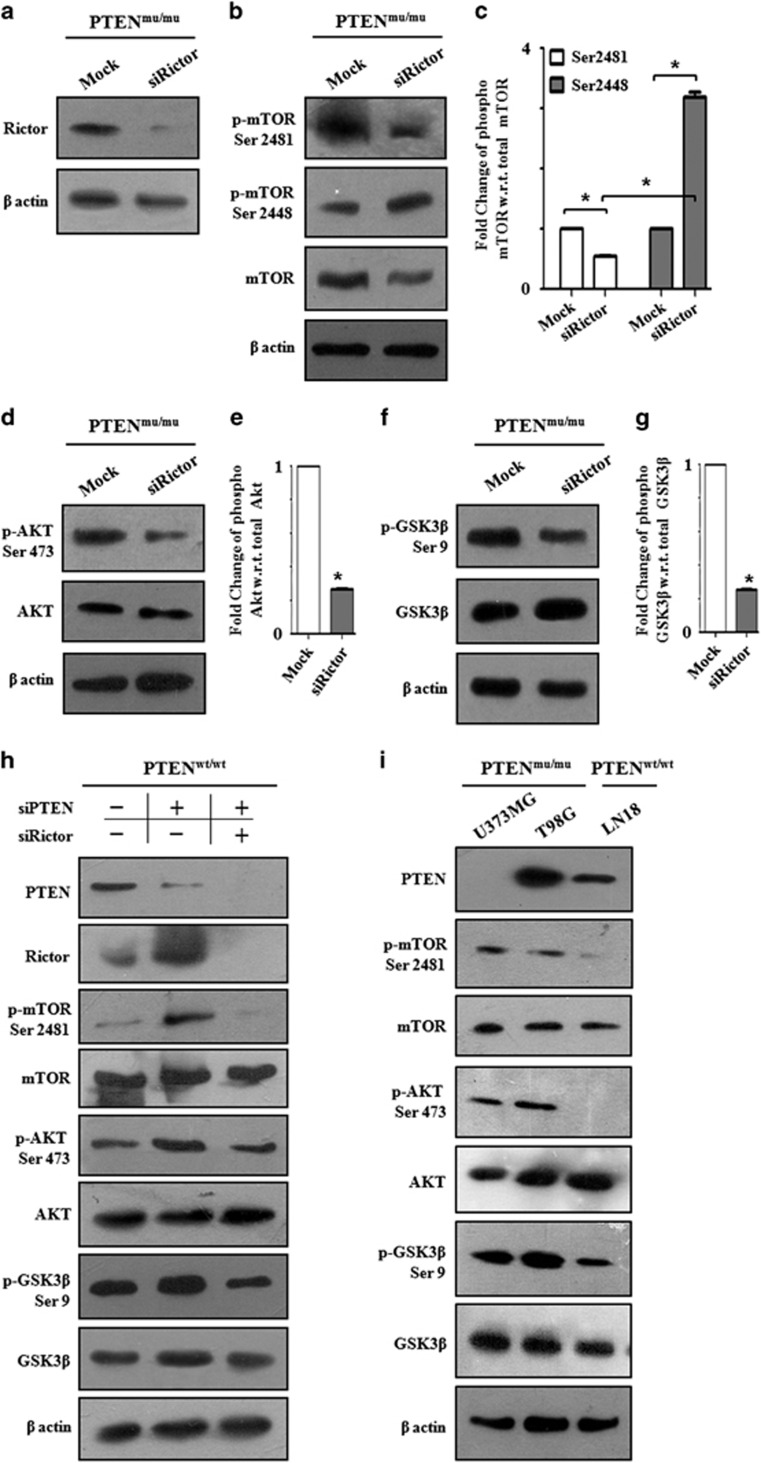
Rictor is essential for mTORC2 signaling upon PTEN mutation. (**a**–**g**) PTEN^mu^ cells were transfected with siRictor. After 24 h of transfection, cell lysates were prepared and subjected for western blot analysis. (**a**) Expression of Rictor was determined**.** (**b**, **c**) Level of mTORC-specific phosphorylations were shown in western blot with relative densitometry values. (**d**, **e**) mTORC2 activity was checked by studying Ser473 phosphorylation of AKT. Relative densitometry analysis of AKT ser473 showed. (**f**, **g**) Status of GSK3β Ser9 phosphorylation with relative densitometric analysis. (**h**) PTEN^wt^ cells were transfected with siPTEN or co-transfected with both siPTEN and siRictor consecutively. After 24 h of transfection, cell lysates were prepared and subjected for western blot analysis. Western blots showed that knockdown of Rictor from PTEN-depleted cell reverted mTORC2 signaling pathway. Single knockdown of PTEN increased phosphorylation of mTOR (Ser2481), AKT (Ser473) and GSK3β (Ser9). Depletion of both PTEN and Rictor decreased phosphorylation of mTOR (Ser2481), AKT (Ser473) and GSK3β (Ser9). (**i**) Status of p-AKT (Ser473), p-GSK3β (Ser9) and p-mTOR (Ser2481) with respect to total protein level in other PTEN^mu^ (T98G, U373MG) and PTEN^wt^ (LN18) GBM cell lines. β-Actin was served as loading control in all set of experiments. All the densitometric analysis were done by ImageJ analysis and normalized against the expression of β-actin and respective total protein level. Each value is the mean±s.d. of three independent experiments. *P<0.05, significant difference between two test groups.

As binding of Rictor to mTOR is the major determining factor for mTORC2 signaling, we wanted to address its role in the PTEN-mutated and/or depleted conditions. Accordingly, we transiently knocked down the expression of Rictor by targeted siRNA in the PTEN^mu^ cells (Figure 3a). Upon Rictor depletion, mTORC2 activation was diminished as evidenced by decreased Ser2481 phosphorylation of mTOR (Figures 3b and c). Subsequently, the specific downstream pathway was also hampered as evidenced by the decreased phosphorylation of Ser473 for AKT (Figures 3d and e) and Ser9 for GSK3β (Figures 3f and g). The relationship between mTORC2 inactivation and corresponding GSK3β activation was also observed after Rictor depletion. This data indicated that upon PTEN deactivation, Rictor is essential for the functional activation of mTORC2 and subsequent downstream signaling.

Next, PTEN^wt^ cells were transfected with siRNA for PTEN or co-transfected with siPTEN and siRictor. Both the single- and double-knockdown of PTEN/Rictor was confirmed (Figure 3h). PTEN knockdown enhanced the overall mTORC2 signaling molecules as observed before (Figure 2). However, when Rictor and PTEN were depleted, the mTORC2 signaling was comparatively reverted. Thus, PTEN knockdown-mediated enhanced phosphorylation of mTOR (Ser2481), AKT (Ser473), and GSK3β (Ser9) were decreased upon Rictor depletion. Therefore, our observation confirmed that Rictor is an essential molecule upon PTEN inactivation for mTORC2 signaling. However, in the absence of Rictor, the basal level phosphorylation of AKT (Ser473) was possibly maintained by AKT autophosphorylation. The literature has shown that AKT can be autophosphorylated (Ser473) by the activation of the AKT kinase domain because of phosphorylation at Thr308.^33^ Therefore, even after depletion of Rictor, a basal level of Ser473 phosphorylation was observed.

We next addressed whether this mechanism was consistent across other GBM cells namely T98G (PTEN^mu^), U373MG (PTEN^mu^) and LN18 (PTEN^wt^). Both PTEN^mu^ cells were hyperactive in mTORC2 signaling as reflected by higher phosphorylation of mTOR (Ser2481) in contrast to PTEN^wt^ (Figure 3i). Downstream substrates (Ser473 of AKT and Ser9 of GSK3β) were also hyperphosphorylated, suggesting that this may be a general phenomenon of PTEN-dependent mTORC2 activation in GBM.

### Functional existence of PTEN negatively regulates mTORC2 complex formation

**Figure 4:**
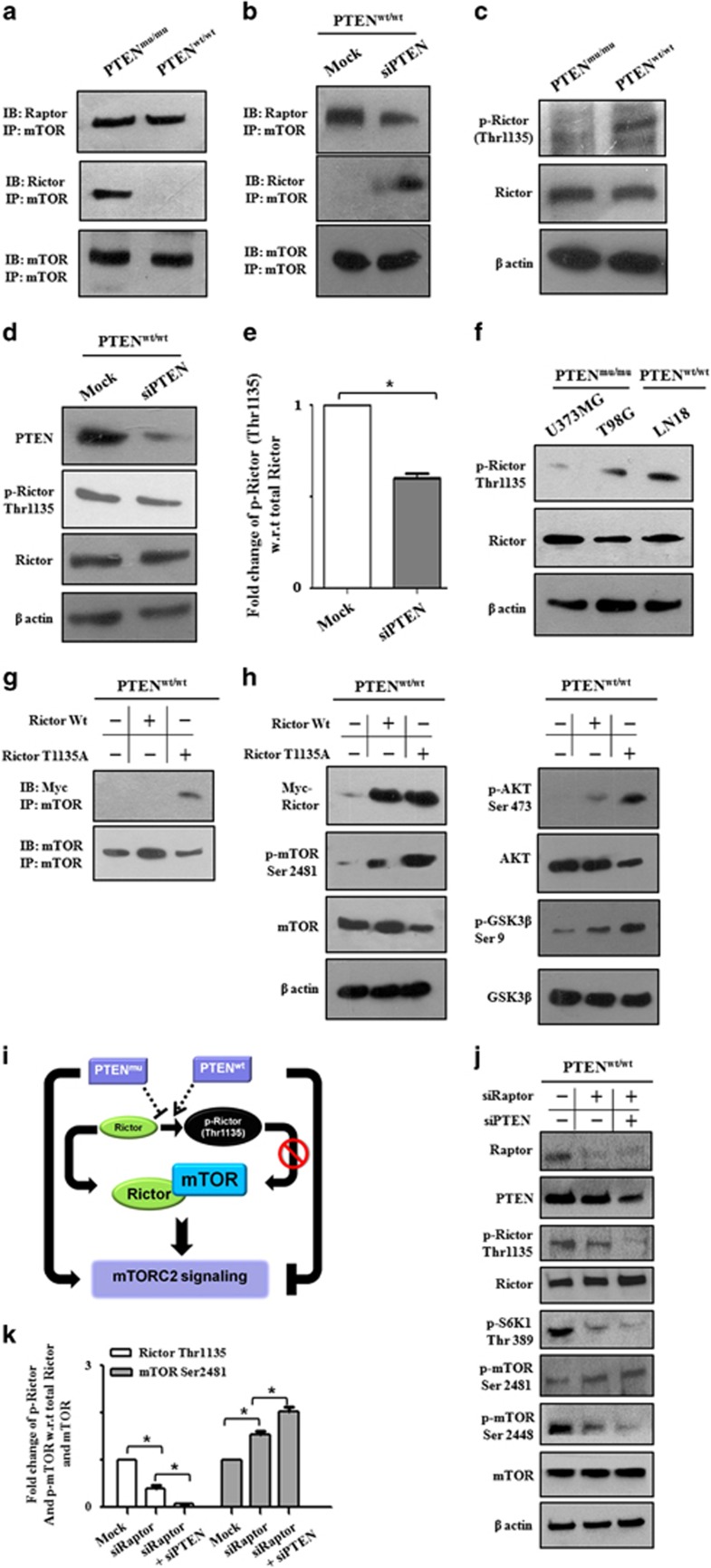
PTEN inhibits mTORC2 formation by hyperphosphorylation of Rictor at Threonine 1135 position. (**a**) PTEN^mu^ and PTEN^wt^ cells were lysed for co-immunoprecipitation (co-IP) study. Protein was incubated with anti-mTOR antibody overnight at 4^o^C followed by incubation with protein A-Sepharose 4B, washed with ice-cold phosphate-buffered saline (PBS). The immunocomplex was resolved by sodium dodecyl sulfate–polyacrylamide gel electrophoresis (SDS–PAGE) and subsequently identified using the respective antibodies. Co-IP revealed that Raptor is associated with mTOR in both the cells, whereas Rictor is associated with mTOR only in PTEN^mu^ cells. (**b**) PTEN^wt^ cells were transfected with siPTEN. After 24 h of transfection, cell lysates were subjected to co-IP analysis. Cells transfected with siPTEN showed increased association of Rictor with mTOR. (**c**) Untreated PTEN^wt^ and PTEN^mu^ cell lysates were subjected for western blot analysis. Expression of phosphorylated Rictor at Thr1135 was detected higher in PTEN^wt^ than PTEN^mu^ cells. (**d**, **e**) PTEN^wt^ cells were transfected with siPTEN. After 24 h of transfection cell lysates were subjected to western blot analysis. Status of Rictor phosphorylation at Thr1135 position was determined. Densitometric analysis showed that there was about 35% reduction of phosphorylation of Rictor (Thr1135) after PTEN knockdown from PTEN^wt^ cells. Each value is the mean±s.d. of three independent experiments. *P<0.05, significant difference between two test groups. (**f**) Other PTEN^mu^ (T98G, U373MG) and PTEN^wt^ (LN18) GBM cell lysates were prepared and subjected for western blot analysis. Status of Rictor Thr1135 phosphorylation was compared. (**g**, **h**) PTEN^wt^ cells (8 × 10^5^) were seeded and incubated for 24 h. Next, cells were transfected with wild-type pRK-5/Rictor (myc-tag) or the T1135A mutant of pRK-5/Rictor (myc-tag, 2 μg/well) or empty vector alone. After 36 h of transfection, cell lysates were prepared and subjected to co-IP (**g**) and western blot analysis (**h**). (**g**) Co-IP showed Rictor was associated with mTOR in cells transfected with pRK-5/Rictor T1135A. (**h**) Immunoblot analysis showed the status of myc-tagged Rictor, p-AKT Ser473 and p-GSK3β Ser9 in those transfected cells. (**i**) Schematic representation: PTEN-mediated regulation of mTORC2 formation and downstream signaling. (**j**, **k**) PTEN^wt^ cells were transfected with siRaptor or co-transfected with siRaptor and siPTEN consecutively. After 24 h of transfection, cells were lysed and subjected to western blot analysis. (**j**) Western blot showed the relative status of mTOR, p-mTOR, p-S6K1, Rictor, p-Rictor Thr1135, AKT, p-AKT Ser473, Raptor and PTEN. (**k**) Densitometry analysis showing the fold change of p-mTOR Ser2481 and p-Rictor Thr1135. Each value is the mean±s.d. of three independent experiments. *P<0.05, significant difference between two test groups. In all the western blot experiments, β-actin served as loading control.

After identifying the importance of Rictor in PTEN-mutated and/or depleted conditions, we asked an obvious question about the interconnection between mTOR complex formation and differential signaling of mTORC2 in PTEN^wt^/PTEN^mu^ cells. A co-immunoprecipitation experiment showed that mTOR was associated with Raptor almost equivalently, indicating a similar level of mTORC1 formation in both cells (Figure 4a). In contrast, mTOR was predominantly associated with Rictor only in PTEN^mu^ cells, demonstrating differential mTORC2 formation between these two cells.

For further reconfirmation, we depleted PTEN from PTEN^wt^ cells using siRNA targeting PTEN and observed a significant association between mTOR and Rictor in deficiency of PTEN, suggesting formation of mTORC2 (Figure 4b). This result suggested that PTEN in active form not only suppresses the mTORC2 signaling but also inhibits the Rictor-dependent mTORC2 complex formation.

### PTEN inhibits mTORC2 formation by stimulating Rictor (Thr1135) phosphorylation

We then addressed the cause of reduced recruitment of Rictor to mTORC in the presence of functional PTEN. Phosphorylation of Rictor (Thr1135) is known to inhibit mTORC2 activity as evidenced by decreased AKT Ser473 phosphorylation.^34^ Accordingly, we addressed whether this phosphorylation was differentially regulated between PTEN^wt^ and PTEN^mu^ cells. Our results revealed that phosphorylation of Rictor at the Thr1135 were significantly higher in PTEN^wt^ cells (Figure 4c). To investigate whether the presence of functional PTEN had any relevance for Rictor Thr1135 phosphorylation, we transiently knocked down PTEN in PTEN^wt^ cells. These knockdown cells exhibited decreased phosphorylation at Thr1135 of Rictor (Figures 4d and e). This result suggested an involvement of PTEN with Rictor hyperphosphorylation at the Thr1135, which in turn possibly inhibits Rictor's association with mTOR, and we observed reduced mTORC2 formation and lower mTORC2 activity. Considering this, we checked the status of Rictor phosphorylation in other PTEN^mu^/PTEN^wt^ cells, and other PTEN^mu^ cells also showed a lower level of Rictor (Thr1135) phosphorylation compared with PTEN^wt^ (Figure 4f), consistent with Figure 3i. These findings support our hypothesis that higher phosphorylation of Rictor is responsible for reduced recruitment to mTORC.

To confirm our findings, we next transfected PTEN^wt^ cells with either wild-type pRK-5/Rictor (myc-tag), the T1135A mutant of pRK-5/Rictor (myc-tag) or the empty vector alone and checked the association of exogenous Rictor and endogenous mTOR by co-immunoprecipitation. Cells expressing mutated Rictor (T1135A) showed enhanced association with endogenous mTOR in comparison with wild-type Rictor/empty vector-transfected cells (Figure 4g). In addition, the level of myc-tag Rictor was equivalent in both wild-type and mutated transfections (Figure 4h). Western blot also revealed that phosphorylation of AKT (Ser473), GSK3β (Ser9) and mTOR (Ser2481) were increased in cells transfected with mutant Rictor (T1135A), indicating higher mTORC2 signaling. These data conclusively established that functional PTEN inhibit mTORC2 formation by promoting Rictor (Thr1135) phosphorylation (Figure 4i).

However, it is unusual that one phosphatase can induce hyperphosphorylation. It has been reported that S6K1 is responsible for the phosphorylation (Thr1135) of Rictor. Earlier, we found that upon PTEN depletion there was an increase in S6K1 phosphorylation (Thr389), that is, the signature of active S6K1 (Figures 2h and i). Accordingly, we hypothesized that upon PTEN depletion in PTEN^wt^ cells, the decrease in Rictor phosphorylation (Thr1135) may revert back because of the increased activity of S6K1. Therefore, the actual effects of PTEN were masked by the increased activity of S6K1. To reveal the definite effects of PTEN depletion upon Rictor phosphorylation, we first inhibited the S6K1 phosphorylation by disrupting active mTORC1 formation, as mTORC1 is responsible for phosphorylation of S6K1. To inhibit mTORC1 activity, we knocked down Raptor in the PTEN^wt^ cells and observed reduced phosphorylation of both S6K1 (Thr389) and mTOR (Thr2448) (Figure 4j). In consequence, the phosphorylation of Rictor (Thr1135) was decreased and mTOR phosphorylation (Thr2481) was increased, indicating S6K1 mediated possible inactivation of mTORC2 (Figures 4j and k). Simultaneous depletion of both Raptor and PTEN from PTEN^wt^ cells triggered a complete reduction in Rictor Thr1135 phosphorylation as well as increased phosphorylation of mTOR (Thr2481). Thus, PTEN depletion coupled with S6K1 deactivation enhances mTORC2 activity through Rictor dephosphorylation (Thr1135).

### Mahanine behaves as a mTORC1/mTORC2 inhibitor, acting differentially in PTEN^mu^ and PTEN^wt^ cells

**Figure 5:**
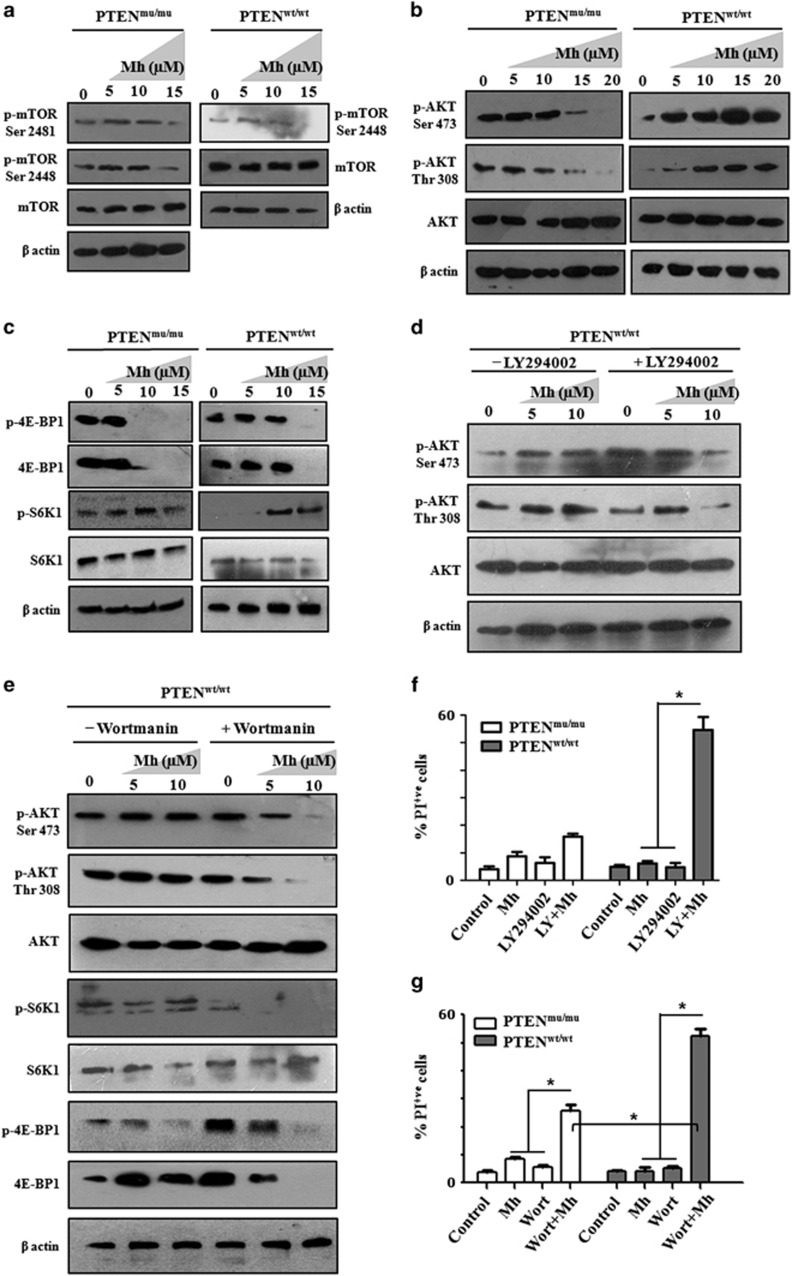
Mahanine, a novel mTORC1/2 inhibitor induces differential downstream regulation of mTORC in PTEN^mu^ and PTEN^wt^ cells. PTEN^mu^ and PTEN^wt^ cells were treated with mahanine (Mh, 5-20 μM) alone or in combination with PI3K inhibitors namely LY294002 (LY, 20 μM) or wortmannin (wort, 100 nM). After 24 h, cells were subjected to western blot and flow cytometry analysis. (**a**) Immunoblot analysis showed expression level of p-mTOR (Ser2448 and Ser2481) in mahanine (0–15 μM) treated PTEN^mu^ and PTEN^wt^ cells after 24 h. Both mTORC1 and mTORC2-specific phosphorylations were disrupted at 15 μM of mahanine. (**b**) Status of AKT activity in mahanine (0–20 μM) treated PTEN^mu^ and PTEN^wt^ cells after 24 h. AKT activity decreased as identified by phosphorylation at the sites of Thr308 and Ser473 in PTEN^mu^ cell. In contrast, both the phosphorylation sites of AKT were hyperphosphorylated in mahanine-treated PTEN^wt^ cells. (**c**) The activity readout of mTORC1 as judged by the phosphorylation of its downstream substrate S6K1 and 4E-BP1 after 24 h of mahanine treatment (0–15 μM) in PTEN^mu^ and PTEN^wt^ cells. (**d**) Status of p-AKT in PTEN^wt^ cell, pre-treated with LY294002 in combination with mahanine. (**e**) Status of S6K1, 4E-BP1 and AKT phosphorylation in PTEN^wt^ cell, pre-treated with PI3K inhibitor (wortmannin) in combination with mahanine. (**f**, **g**) PTEN^mu^ and PTEN^wt^ cells were treated with different doses of mahanine (0–15 μM), LY294002 (0–30 μM) and wortmannin (50–150 nM) alone or in combination with mahanine–LY294002 or mahanine–wortmannin. After 24 h of treatment, dead cell populations were determined by PI staining. (**f**) Mahanine (12 μM) in combination with LY294002 (20 μM) showed synergistic cell death activity in PTEN^wt^ cells. (**g**) Mahanine (10 μM) in combination with wortmannin (100 nM) showed synergistic cell death activity in PTEN^wt^ cells. Each value is the mean±s.d. of three independent experiments. *P<0.05, significant difference between two test groups.

Identification of differential mTORC signaling was the foundation for the development of chemotherapeutic agents. We previously identified mahanine as a potential chemotherapeutic agent for GBM (Supplementary Figure S1).^25^ It displayed a cytotoxic effect against PTEN^mu^ and PTEN^wt^ cells (Supplementary Figure S2). Here we observed that mahanine suppressed both Ser2448 and Ser2481 phosphorylation of mTOR in PTEN^mu^ cells, whereas only Ser2448 phosphorylation was inhibited in PTEN^wt^ cells (Figure 5a). Owing to the low level of Ser2481 phosphorylation of mTOR in PTEN^wt^ cells, mahanine-mediated regulation was almost undetectable. This result indicated that mahanine act as both an mTORC1 and mTORC2 inhibitor in PTEN^mu^/PTEN^wt^ cells, depending on the existence of those complexes.

To confirm this mTOR-inhibitory mechanism of mahanine, we studied its downstream AKT pathway. Mahanine-treated PTEN^mu^ cells exhibited decreased phosphorylation at Thr308 and Ser473 when both mTORC1 and mTORC2 were present (Figure 5b). However, mahanine behaved similarly in both PTEN^mu^ and PTEN^wt^ by inducing downregulation of 4E-BP1 in total protein level and phosphorylated forms but phosphorylation of S6K1 (Thr389) was decreased only in PTEN^mu^ cells in contrast to PTEN^wt^, where phosphorylation at this site was increased (Figure 5c).

Although mahanine showed cytotoxicity in PTEN^wt^ cells, we did not observe any inhibition of AKT phosphorylation (Thr308 and Ser473) when mTORC2 was not in significant level and mainly mTORC1 was present (Figure 5b). Therefore, mahanine was possibly inducing cell death in PTEN^wt^ cells by targeting other pathways. Inhibition of mTORC1 releases the feedback inhibition mediated by the S6K1-IRS1-PI3K loop, which leads to activation of the AKT pathway in cancer.^35^ Accordingly, we inhibited the PI3K pathway using two specific inhibitors, wortmannin and LY294002, separately to check the status of AKT activation in mahanine-treated PTEN^wt^ cells. Mahanine, even at a low dose, exhibited reduced AKT phosphorylation at Thr308 and Ser473 sites in the presence of these inhibitors, suggesting involvement of the PI3K pathway in AKT activation in PTEN^wt^ cells (Figures 5d and e). We also checked other downstream effector molecules of mTORC1 and observed phospho-S6K1 (Thr389) and 4E-BP1 (Thr37/46) were reduced significantly when the mTORC1/2 inhibitor was used in combination with wortmannin as compared with the inhibitor alone (Figure 5e). Furthermore, the mTORC1/2 inhibitor with LY294002 or wortmannin synergistically enhanced the cell death in PTEN^wt^ cells compared with the inhibitor alone (Figures 5f and g). Overall, we demonstrated that, in the PTEN^wt^ condition, where mTORC1 formation was predominant and mTORC2 was not present in significant amount, this mTORC1/2 inhibitor behaved like an mTORC1 inhibitor and enhanced the AKT-mediated cell protective pathway, which could be reverted by PI3K inhibition.

### A therapeutic strategy to treat GBM by suppressing native PTEN to enhance the activity of the mTORC1/2 inhibitor

**Figure 6:**
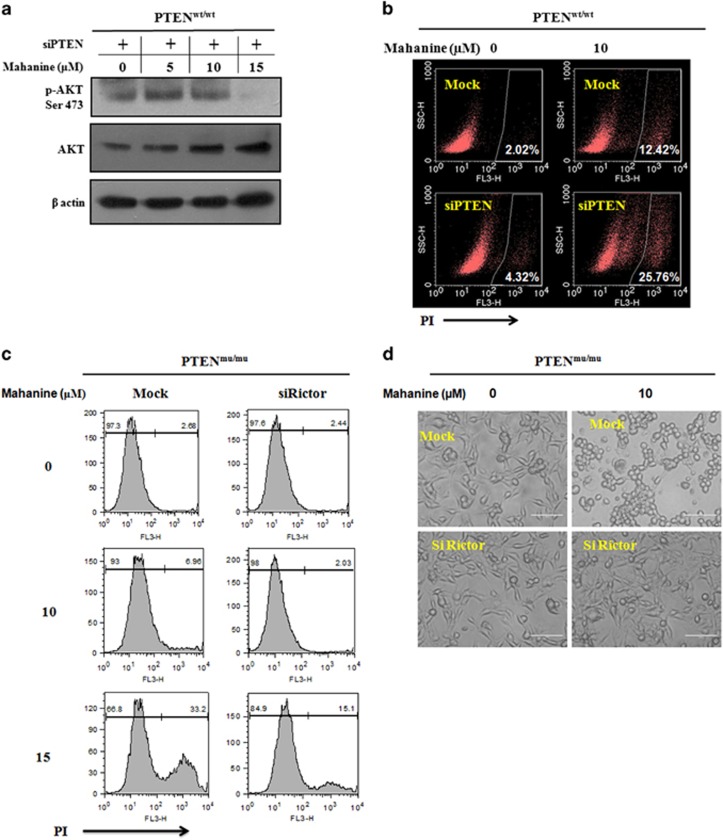

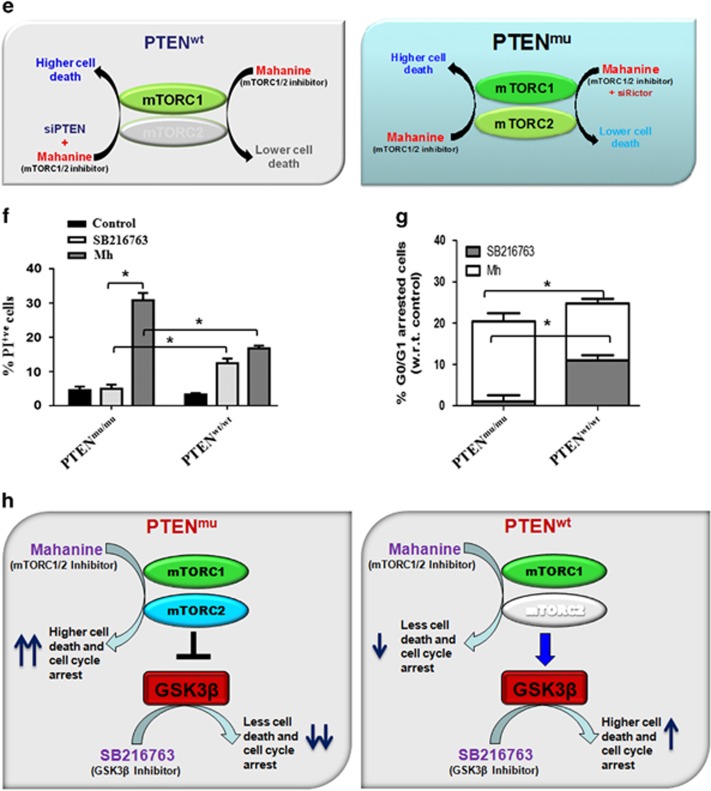
Differential sensitivity of mTORC1/2 inhibitor and GSK3β inhibitor in PTEN^wt^ and PTEN^mu^ cells based on the existence of mTORC2. (**a**, **b**) PTEN^wt^ cells were transfected with siPTEN and after 24 h, they were treated with mahanine (0–15 uM). (**a**) The expression levels of phospho-AKT Ser473 were revealed by western blotting. (**b**) Dead cell population was determined by PI staining. (**c**, **d**) PTEN^mu^ cells were transfected with siRictor and after 24 h, they were treated with mahanine (0–15 μM). (**c**) Mahanine-mediated cell death was determined by PI-positive cells by flow cytometric analysis. (**d**) Light microscopic image showed the morphology of PTEN^mu^ cells after mahanine treatment. (**e**) Schematic diagram showed possible strategy to enhance the activity of mTORC1/2 inhibitor in GBM. (**f**, **g**) PTEN^mu^ and PTEN^wt^ cells were treated with mahanine (12 μM) and GSK3β inhibitor (SB216763, 20 μM). After 24 h of treatment, cells were processed for cell cycle analysis. Dead cell population was determined by PI staining by flow cytometry. Each value is the mean±s.d. of three independent experiments. *P<0.05, significant difference between two test groups. (**h**) Schematic representation: reciprocal responsiveness of mahanine and GSK3β inhibitor depending on existence of mTORC2 in PTEN^wt^ and PTEN^mu^ cells.

Next, we addressed the role of PTEN in activation of AKT in mahanine-treated PTEN^wt^ cells. We depleted PTEN from the PTEN^wt^ cells and observed the effect of the mTORC1/2 inhibitor. Western blot analysis revealed that the mTORC1/2 inhibitor could inhibit AKT activation, identified by a reduction in phosphorylation in PTEN^wt^ cells when the PTEN was depleted by siRNA (Figure 6a). The activity of the mTORC1/2 inhibitor in PTEN-depleted PTEN^wt^ cells was enhanced, as identified by the increased propidium iodide (PI) positivity compared with mock-transfected cells (Figure 6b). This is possibly because PTEN depletion induces generation of mTORC2 as a target complex. This result indicated that the mTORC1/2 inhibitor exhibited more effects when PTEN is mutated and subsequently mTORC2 is activated.

To confirm our findings, we next inhibited mTORC2 formation in PTEN^mu^ cells by depleting Rictor and then treated with mahanine. We observed decreased PI positivity when there was a reduction in mTORC2 activity (Figure 6c). Microscopic images also proved that the mTORC1/2 inhibitor has a significantly lowered cytotoxic effect after Rictor knockdown (Figure 6d). Therefore, we concluded that cells having enhanced mTORC2 are more sensitive to the mTORC1/2 inhibitor. Our overall findings established that, as in PTEN^wt^ cells, no significant mTORC2 formation were occurring, so the mTORC1/2 inhibitor-induced AKT activity was driven by the sole inhibition of mTORC1. Upon PTEN depletion, they behave like a dual mTORC1/2 inhibitor because of the formation of enhanced mTORC2 as a target molecule (Figure 6e). On the contrary, Rictor depletion inhibits mTORC2 formation, made PTEN^mu^ cells less sensitive to the mTORC1/2 inhibitor. Thus, PTEN has an important role in mTORC2 formation and also influences the effect of an mTORC1/2 inhibitor in GBM.

### mTORC2 formation showed reduced sensitivity to a GSK3β inhibitor

Along with mTORC1/2, GSK3β has long been a chemotherapeutic target in GBM. We had identified a significant reciprocal activation between mTORC2 and GSK3β (Figures 1g and 2f). In PTEN^mu^ cells, mTORC2 was activated and GSK3β was inactivated, whereas in PTEN^wt^ cells the situation was the reverse. To check the functionality of this observation, we performed a cell death assay and observed that a specific GSK3β inhibitor (SB216763) showed lower PI positivity, indicating lower cell death in PTEN^mu^ cells compared with PTEN^wt^ (Figure 6f). We hypothesized that in PTEN^mu^ cells, GSK3β was already inhibited by a higher level phosphorylation (Ser9) and therefore the addition of a GSK3β-inhibitor did not exert any extra effect. In contrast, an mTORC1/2 inhibitor (mahanine) exhibited significantly enhanced cytotoxicity in PTEN^mu^ cells. This was further confirmed by demonstrating higher G0/G1 phase cell cycle arrest (Figure 6g). These results validated that, depending on PTEN status, mTORC2 and GSK3β maintain a reciprocal relationship. Cells, which are susceptible to mTORC2 inhibition, may not be susceptible to GSK3β inhibition and vice versa (Figure 6h).

## Discussion

Mutation of PTEN is associated with high-grade GBM formation. mTORC2 is emerging as a potential drug target in PTEN-mutated cancers. The detailed mechanism of molecular regulation and cross-talk between PTEN and mTORC2 is still not clearly known. Accordingly, we investigated the role of PTEN in mTORC2 regulation and signaling in GBM. The major achievement of this study is providing evidence that PTEN^mu^ cells exhibit enhanced mTORC2 formation and signaling because of the decreased inhibitory phosphorylation of Rictor (Thr1135). This is the first evidence suggesting Thr1135 phosphorylation of Rictor is controlled by PTEN, and demonstrates PTEN as the master regulatory molecule in mTORC2 formation. In addition, we have established mahanine, carbazole alkaloid from an edible plant, as a potential mTORC1/2 inhibitor that showed cytotoxicity both in PTEN^mu^/PTEN^wt^ cells.

mTORC2 activity is required for generation of PTEN deletion-induced prostate cancer.^36^ EGFRvIII-mediated activation of mTORC2 was reversed by PTEN activity.^37^ A correlation between PTEN and Rictor was also reported in leukemogenesis.^38^ Here we showed that PTEN^mu^ cells have higher mTORC2 formation and downstream signaling than PTEN^wt^ cells as evidenced by enhanced mTOR Ser2481, AKT Ser473 and GSK3β Ser9 phosphorylations. Although a correlation between PTEN and AKT Ser473 phosphorylation was reported in lung cancer and rhabdomyosarcomas, no cross-linking molecule between these phosphatase and kinase was demonstrated.^39, 40^ Here, we showed mTORC2 is a connecting molecule between PTEN and AKT.

During exploration of mTORC1/2-associated molecules, we observed that Rictor is differentially phosphorylated (Thr1135) with respect to the functional existence of PTEN. Rictor phosphorylation (Thr1135) is responsible for decreased activity of mTORC2 and reduced AKT (Ser473) phosphorylation.^34^ Therefore, reduced mTORC2 activity and AKT Ser473 phosphorylation in PTEN^wt^ cells is a consequence of increased Rictor Thr1135 phosphorylation. This event was confirmed when we knocked down PTEN in PTEN^wt^ cells and observed reduced Rictor phosphorylation, and as a consequence, enhanced mTORC2 signaling. Although Rictor Thr1135 phosphorylation is responsible for decreased activity of mTORC2, reports suggest this had no effect on mTORC2 complex formation.^34, 41, 42, 43^ However, the detailed mechanism behind reduced mTORC2 activity without hampering mTORC2 complex formation was not revealed. Our data revealed that not only was downstream signaling affected, but also there was differential mTORC2 complex formation in PTEN^wt^ and PTEN^mu^ cells. PTEN^wt^ cells lack significant mTORC2 formation, and that was reverted with PTEN knockdown. Therefore, we conclude that in the presence of PTEN^wt^, there is enhanced Rictor Thr1135 phosphorylation, decreased mTORC2 formation, which connected to lower AKT activity, and increased GSK3β activity. The role of PTEN in regulating Thr1135 phosphorylation and mTORC2 complex formation was further demonstrated by expressing T1135A mutant Rictor in PTEN^wt^ cells. We observed significant mTORC2 complex formation and downstream signaling in the presence of PTEN when there was a mutant Rictor that cannot be phosphorylated on Thr1135. Therefore, we concluded that this may be a unique phenomenon in GBM where phospho-Rictor Thr1135 could not be recruited to mTORC2 in the presence of PTEN^wt^.

Thr1135 phosphorylation of Rictor is also controlled by S6K1 via mTORC1.^34,43^ This downstream kinase is known to be a linker molecule between mTORC1/2. Therefore, we checked mTORC1 activity and found equivalent mTORC1 formation and downstream activity as judged by comparable phosphorylation of downstream signaling molecules (S6K1 and 4E-BP1). This data reconfirmed the involvement of PTEN in regulation of mTORC2 in GBM. Therefore, S6K1 may not be solely responsible for controlling Rictor (Thr1135) phosphorylation. Simultaneous suppression of S6K1 activity and functional PTEN in PTEN^wt^ cells completely abolishes Rictor phosphorylation (Thr1135) and enhances active mTORC2 formation. However, PTEN, being a phosphatase, cannot phosphorylate a molecule directly. We assume that PTEN may be affecting some other kinases by removing inhibitory phosphorylation, and they in turn phosphorylate Rictor at the specific site. Alternatively, it may also be possible that PTEN dephosphorylates some other site of Rictor that enables the molecule to change its conformation in such a way so that the Thr1135 site is exposed for further phosphorylation by known kinases like S6K1. Experiments to distinguish between these possibilities are ongoing.

Basic understandings at the cellular and molecular level are successful when a translational or therapeutic approach works out positively. We have observed mahanine exerted cytotoxicity both in PTEN^mu^ and PTEN^wt^ cells thus behaved as an mTORC1/2 inhibitor. However, in PTEN^wt^ cells, where mTORC2 was not significantly active and mTORC1 is predominant, mahanine behaves like an mTORC1 inhibitor. Here we observed an activation of the AKT pathway in PTEN^wt^ cells. Several reports indicated that mTORC1 inhibition could lead to activation of AKT by other pathways.^35, 44, 45^ This was due to release of the negative feedback loop via S6K1 leading to the activation of PI3K/AKT pathway.^44^ mTORC1 inhibition also leads to MAPK pathway-dependant AKT activation.^45^

This activation of AKT was reversed in PTEN^wt^ cells in the presence of specific PI3K inhibitors. We also observed a synergistic effect when a combination of mTORC1/2 and PI3K inhibitors were used to overcome this AKT activity in PTEN^wt^ cells. These data suggest that for an effective therapeutic strategy both mTORC1/2 and PI3K inhibitors may be necessary for a beneficial effect in PTEN^wt^ GBM.

Our findings demonstrated that PTEN behaved like a physiological inhibitor of mTORC2 formation. Generation of mTORC2 upon PTEN depletion provided the specific target of mahanine and hence cell death was increased in PTEN^wt^ cells.

We have observed a reciprocal relationship between mTORC2 and GSK3β in GBM. Therapeutically, we also proved this reciprocal relationship. In PTEN^mu^ cells where mTORC2 and AKT are active and GSK3β is inactive, treatment with GSK3β inhibitors did not add any extra benefit. In contrast, a GSK3β inhibitor exerted a significant effect on PTEN^wt^ cells where GSK3β is active.

Studies have suggested that mTORC1/C2 inhibitors have more effect on PTEN-mutated cancers.^46, 47^ Our results convincingly showed the mechanism whereby PTEN mutations increase the sensitivity of cancers to mTORC1/2 inhibitors. We have established loss of PTEN induces more mTORC2 formation, and more susceptible to mTORC1/2 inhibitors. This hypothesis was further proven when we depleted Rictor from PTEN^mu^ cells, which had abundant mTORC2 formation. Depletion of Rictor leads to decreased mTORC2 formation, and therefore they behaved like PTEN^wt^ cells, with predominant mTORC1. Accordingly, mTORC1/2 inhibitors acted as only mTORC1 inhibitors and we observed a reduction in cell death. Therefore, mTORC2 is the prime factor that is responsible for differential sensitivity to mTORC1/2 inhibitors. PTEN showed its effect by inhibiting Rictor and thereafter mTORC2 kinase activity in PTEN^wt^ cells.

This observation was additionally confirmed by using a well-known mTORC1 inhibitor, rapamycin. Although rapamycin shows cytotoxicity in PTEN^wt^/PTEN^mu^ cells, however, these cells activate AKT as evidenced by the enhanced Thr308 and Ser473 phosphorylation. Phosphorylation of 4E-BP1 remained unchanged whereas S6K1 was decreased (Supplementary Figure S5). Therefore, these data indicate again that not only mahanine but other mTORC1 inhibitors also activate AKT in PTEN^wt^ cells. Most importantly, a sole mTORC1 inhibitor can hyperphosphorylate AKT irrespective of PTEN status.

To correlate our observation, we found that mahanine inhibited AKT in two PTEN^mu^ prostate cancer cells.^26, 32^ Mahanine, in contrary, prevented palmitate-induced inhibition of the insulin-stimulated phosphorylation of AKT in a PTEN^wt^ cell.^48^ According to our hypothesis and findings, in PTEN^mu^ prostate cancer cells, mahanine might inhibit AKT completely by suppressing both mTORC1/2. In contrast, mTORC2 could not form in the PTEN^wt^ (L6) cells and mahanine behaved like an mTORC1 inhibitor and enhanced AKT activity by the inhibition of a negative feedback loop.

Therefore, it is envisaged that PTEN-dependent mTORC1 and mTORC2 dynamics are not restricted only to the GBM system and could be a global phenomenon. Our findings will open up a new avenue of thought for designing modified and efficient therapy against malignancy. However, more in-depth studies are needed to uncover the molecular function of PTEN in regulation of mTORC2.

## Materials and methods

### Reagents

All the antibodies were from Cell Signaling Technology (Danvers, MA, USA): mTOR (cat #2983), phospho-mTOR (Ser2481, cat #2974), phospho-mTOR (Ser2448, cat #5536), Rictor (cat #9476), phospho-Rictor (Thr1135, cat #3806), Raptor (cat #2280), AKT (cat #4691), phospho-AKT (Ser473, cat #4060), phospho-AKT (Thr308, cat #13038), phospho-AKT (Thr450, cat #9267), GSK-3β (cat #9315), phospho-GSK-3β (Ser9, cat #9336), PTEN (cat #9559), β-actin (cat #4970), 4E-BP1 (cat #9644), phospho-4E-BP1 (Thr37/46, cat #2855), S6K1/p70S6K (cat #9202), phospho-S6K1/phospho-p70S6K (Thr389, cat #9234), Myc-Tag (cat #2276), horseradish peroxidase-linked anti-rabbit IgG (cat #7074), horseradish peroxidase-linked anti-mouse IgG (cat #7076). Lipofectamine LTX, plus reagent, culture medium were from Invitrogen (Carlsbad, CA, USA). Antibiotic–antimycotic, propedium iodide (PI), rapamycin, GSK3β inhibitor (SB212763), PI3K inhibitor (LY294002 and wortmannin) and other chemicals were from Sigma–Aldrich (St Louis, MO, USA). Protease and phosphatase inhibitor cocktails were from Calbiochem (San Diego, CA, USA). Cycle Test Plus kit was from BD Bioscience (East Rutherford, NJ, USA). Super Signal West Pico imaging system was from Thermo Scientific (Rockford, IL, USA).

### Cell lines and culture condition

PTEN^mu^ (U87MG, T98G and U373MG) and PTEN^wt^ (LN229 and LN18) human GBM cell lines (Supplementary Figure S4) were from ATCC (Manassas, VA, USA) and mycoplasma contamination was checked. They were cultured in Iscove's modified dulbecco's medium (IMDM) supplemented with 10% heat inactivated fetal bovine serum and 1% antibiotic–antimycotic, and maintained at 37 ^o^C with 5% CO_2_. For specific experiments, cells (1 × 10^4^−1 × 10^6^) were treated with various inhibitors/drugs under identical conditions.

### Transfection, siRNA and plasmids

siRNA against human PTEN, Rictor and Raptor were from Ambion Life Technologies, Carlsbad, CA, USA (Supplementary Figure S3). Vector encoding myc-tag wild-type Rictor (pRK-5/Rictor) or T1135A mutated form of Rictor (pRK-5/Rictor T1135A) was kind gift from Dr Philippe P Roux (Faculty of Medicine, Universite' de Montre'al, Montreal, Quebec, Canada). PTEN^mu^/PTEN^wt^ cells were transiently transfected with human PTEN/Rictor/Raptor targeting siRNAs (Supplementary Figure S3) or pRK-5/Rictor or pRK-5/Rictor T1135A or empty vector alone using lipofectamine LTX and plus reagent following the manufacturer's instruction. PTEN-depleted/Rictor-depleted cells were further treated with inhibitors for 24 h and processed for western blot and flow cytometric analysis.^25^

### Immunoblotting

Drug/inhibitor treated or siRNA/plasmid transfected or untreated/untransfected cells were harvested and lysed and processed for western blot analysis.^25^ Target proteins were identified by the West Pico chemiluminiscence detector system or Biorad ChemiDoc MP System (Bio-Rad, Hercules, CA, USA). Developed bands were densitometrially analyzed by ImageJ software (NIH, Bethesda, MD, USA).^25^

### Co-immunoprecipitation and immunoprecipitation

For detection of the mTOR complexes, cells were lysed in ice by sonication (Qsonica-LLC, XL-2000 series, Newtown, CT, USA). Protein (200 μg) was incubated with anti-mTOR antibody (1:100) overnight at 4 ^o^C. Immunocomplex was incubated with protein A-sepharose 4B, washed with ice-cold phosphate-buffered saline, resolved by sodium dodecyl sulfate–polyacrylamide gel electrophoresis and subsequently identified using the respective antibodies.

### Detection of cell death and cell cycle analysis by MTT assay and flow cytometry

Mahanine was purified as described previously.^31^ Cell viability of mahanine (0–30 μM)-treated cells (1 × 10^4^) was checked after 48 h. They were further incubated with MTT (100 μg/ml) for 3 h. Formazan crystals were dissolved in dimethylsulfoxide and optical density was taken at 550 nm.^25^ Cells (1 × 10^6^) were treated with mahanine, SB216763, Wortmanin and LY294002 alone or in combination for 24 h and processed using Cell Cycle Test Plus kit.^25^ At least, 20 000 cells were acquired and analyzed by flow cytometry using CellQuest Pro software (BD FACSCalibur, San Jose, CA, USA).^49^ Morphology of mahanine-treated cells were imaged by transmitted light digital inverted microscope (EVOS XL Core, Life Technology, Carlsbad, CA, USA).

### Statistical analysis

All the data were from at least three independent experiments and statistical analysis was performed using Graph Pad Prism 5 (La Jolla, CA, USA). The differences between the groups were analyzed by two tail Student's *t*-test or Mann–Whitney *U*-test. Standard error bars represent the s.d. of the mean (±s.d.) and *P<0.05, denoted the significant differences between the means of the untreated and treated cells or two test groups.
