# Supplementary figures and images for: PTEN negatively regulates mTORC2 formation and signaling in grade IV glioma via Rictor hyperphosphorylation at Thr1135 and direct the mode of action of an mTORC1/2 inhibitor
Source: Oncogenesis. 2016 May 30;5(5):e227–. doi: 10.1038/oncsis.2016.34 (PMC4945751; doi:10.1038/oncsis.2016.34)

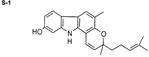

Supplement: Supplementary Figure 1 [file oncsis201634x1.tif]

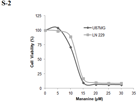

Supplement: Supplementary Figure 2 [file oncsis201634x2.tif]

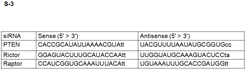

Supplement: Supplementary Figure 3 [file oncsis201634x3.tif]

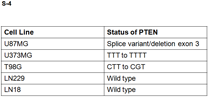

Supplement: Supplementary Figure 4 [file oncsis201634x4.tif]

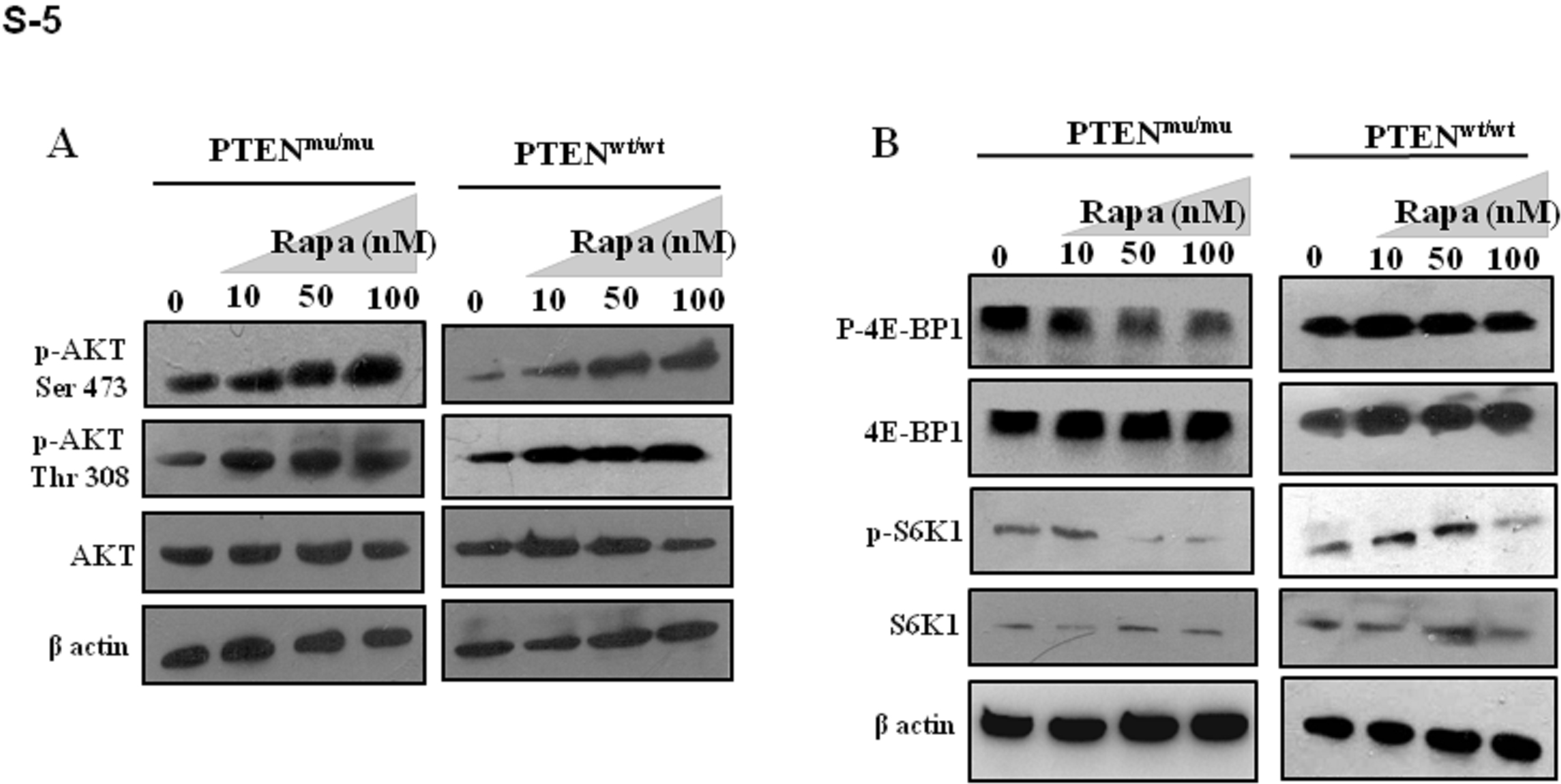

Supplement: Supplementary Figure 5 [file oncsis201634x5.tif]
