# Supplementary material for: PTEN negatively regulates mTORC2 formation and signaling in grade IV glioma via Rictor hyperphosphorylation at Thr1135 and direct the mode of action of an mTORC1/2 inhibitor
Source: Oncogenesis. 2016 May 30;5(5):e227–. doi: 10.1038/oncsis.2016.34 (PMC4945751; doi:10.1038/oncsis.2016.34)
Supplement: Supplementary Information [file oncsis201634x6.docx]

**S-1.** Chemical structure of mahanine isolated from *Murraya koenigii.*

**S-2.** Mahanine induced decrease cell viability (%) as measured by MTT assay after 48 hours.

**S-3.** Information and sequence of siRNA specific for PTEN, Rictor and Raptor

**S-4.** Status of PTEN mutation in different GBM cell lines used in this study.

**S-5.** PTEN^mu^ and PTEN^wt^ cells were treated with rapamycin (0-100 nM) for 24 hours. Cell lysates were subjected for immunoblot analysis and probed against indicated antibodies.
